# Neuropsychiatry revisited: epilepsy as the borderland between neurology and psychiatry

**DOI:** 10.3389/fpsyt.2024.1486667

**Published:** 2024-09-27

**Authors:** Daichi Sone, Kousuke Kanemoto

**Affiliations:** ^1^ Department of Psychiatry, Jikei University School of Medicine, Tokyo, sJapan; ^2^ Department of Neuropsychiatry, Aichi Medical University, Nagakute, Japan

**Keywords:** epilepsy, neuropsychiatry, comorbidity, psychosis, depression, cognitive dysfunction, neurodevelopmental disorders

## Abstract

Since epilepsy is often complicated by psychiatric symptoms, the contributions of psychiatry are indispensable for the care and improvement of the quality of life of individuals with epilepsy. Moreover, the existence of a bidirectional relationship between epilepsy and psychiatric symptoms was recently proposed, based on the evidence that not only are some psychiatric symptoms more likely than others to follow epilepsy, but also that psychiatric symptoms may precede the onset of epilepsy and the presence of psychiatric symptoms may influence the outcome of treatment for seizures. There has also been a gradual accumulation of neurobiological findings related to psychosis, depressive, and anxiety symptoms that are associated with epilepsy with respect to abnormalities in brain networks and neurotransmission. This mini-review focuses on the neuropsychiatric aspects of epilepsy and proposes that a reconsideration of neuropsychiatry in light of epilepsy findings could serve as a bridge between psychiatry and neurology.

## 1 Introduction

Neuropsychiatry has been described as a field of medicine “in which neurology is relevant in understanding mental and behavioral illness” “that is concerned with the complex relationship between human behavior and brain function and understanding abnormal behavior and behavioral disorders on the basis of neurobiological and psychosocial factors” (1, 2). While mental phenomena may not always be reduced to neural phenomena, neuropsychiatry considers the mind as an emergent property of the brain and views mental disorders as disorders of the brain (1). Neuropsychiatry may be regarded as a specialty that integrates psychiatry, neurology, and neuropsychology (2). Historically, the fields of neurology and psychiatry have interacted with each other, but they are currently practiced in independent clinical departments despite a certain amount of overlap. A thorough knowledge of neurological diseases and symptoms is still important in clinical psychiatry, as individuals with neurological diseases that involve the brain can present various psychiatric and behavioral symptoms. Similarly, neuropsychiatry or behavioral neurology may help neurologists to understand and treat neurological disorders presenting behavioral symptoms. This mini-review discusses the neuropsychiatric aspects of epilepsy.

## 2 Epilepsy and neuropsychiatry: a bidirectional relationship and beyond

The Greek verb “epilambanein,” from which the word “epilepsy” is derived, means “to be seized,” which implies the experience of being seized by a being that is beyond human understanding (3). In the Middle Ages, the dominant belief was that evil spirits or a moon curse were responsible for epilepsy, but the work by the British neurologist John Hughlings Jackson (1835–1911) and the introduction of electroencephalography (EEG) developed the current concept of epilepsy as a brain disorder caused by recurrent, abnormal, excessive or synchronized electrical discharges (3, 4).

However, in addition to the high prevalence of psychiatric comorbidities in epilepsy, evidence described in 2012 suggests a bidirectional relationship between epilepsy and psychiatric symptoms (5). The lifetime prevalence of psychiatric symptoms among individuals with epilepsy is reported to be 35% (6), with an odds ratio 2–5 times higher than that of people without epilepsy (7, 8). This lifetime prevalence is higher than those of other chronic diseases, such as bronchial asthma (9), and it thus may not be attributed solely to the presence of a chronic disease. On the other hand, on the basis of a bidirectional relationship, psychiatric symptoms are also known to influence epileptic seizures. For example, depression and anxiety symptoms sometimes precede the onset of epilepsy, and depression worsens the seizure prognosis following pharmacotherapy and surgical treatments (5). Further, the therapeutic effects of electroconvulsive therapy on psychiatric symptoms (10) could be argued in favor of a bidirectional relationship between psychiatric symptoms and epilepsy.

However, a bidirectional relationship means nothing more than “not a one-way relationship,” and simply stating that it is bidirectional does not reveal anything more than that. Further research is necessary regarding several putative mechanisms such as brain structural and functional changes, neurotransmitters, neuroimmunity, and the endocrine system (5) in order to clarify the actual pathological relationship between epilepsy and psychiatric symptoms.

## 3 Psychiatric, behavioral, and cognitive symptoms in epilepsy

In addition to a classification by symptom content as is applied in other psychiatric disorders, psychiatric comorbidities among patients with epilepsy are also classified according to their temporal relationship to seizures. Pre-ictal symptoms (which occur ~1–2 days before a seizure), ictal symptoms (which correspond to a seizure itself), and post-ictal symptoms (which occur within a few weeks after the seizure[s]) are called peri-ictal symptoms, and they are distinguished from symptoms occurring during the interictal period (11, 12). The postictal phase refers to a transient brain condition within minutes to days after seizures, which manifests various neurological deficits and/or psychiatric symptoms (13). Psychiatric symptoms may also appear with the suppression of seizures and the normalization of EEG findings, and the concepts of forced normalization and alternative psychosis have been proposed, although they remain controversial (11).

Categories based on symptom content include cognitive dysfunction, psychotic symptoms, mood and anxiety symptoms, and personality changes and traits (14). The aforementioned associations of such symptoms with seizures and EEG results as well as a causal relationship with antiseizure medication therapy are considered next.

## 4 Epilepsy and psychosis

The odds ratio for the appearance of psychosis in epilepsy is reported to be 7.8 times higher than in the general population (15). In postictal psychosis (11), a psychotic state with rapid agitation, confusion, and emotional coloration typically appears within a week after a seizure (especially in clusters) and disappears within a few weeks. This is often accompanied by an asymptomatic period called the lucid interval, which lasts 1–2 days after the seizure. Postictal psychosis is particularly common among individuals with temporal lobe epilepsy, at >10 years after the epilepsy’s onset. In contrast, interictal psychosis is not necessarily present in temporal lobe epilepsy and tends to occur ~15 years after the onset of epilepsy. The symptoms are more similar to those of schizophrenia, but the negative symptoms are less prominent (16).

Although several research groups have investigated the neural basis of psychotic symptoms in epilepsy, the mechanisms have not been elucidated in part because of the relatively inconsistent findings (17). Atrophy of the posterior hippocampus (18), abnormal brain network function (19, 20), increased glucose metabolism (21), and abnormal brain aging (22, 23) have been suggested, but further validation and elucidation of the biological bases is desired.

## 5 Epilepsy and depression/anxiety

It has been repeatedly reported that people with depressive disorders are at higher risk of developing epilepsy (5), which might be a sign of prodromal brain before the first epileptic seizure occurs. Depressive symptoms in epilepsy may affect an individual’s quality of life more than seizures (24) and may also worsen seizure outcomes after pharmacotherapy and surgical treatment (5). Some groups have proposed a dysphoric disorder characterized by irritability, depression, pain, anxiety, insomnia, and euphoria (25), but this may not be specific to epilepsy (26) and remains controversial. In terms of treatment, only one small double-blind, placebo-controlled randomized controlled trial (RCT) of antidepressants for depression among patients with epilepsy reported no superiority to placebo (27, 28). There have been few similar RCTs are (29, 30), and evidence is lacking as to whether patients with epilepsy can be treated in the same way as patients with major depressive disorder without epilepsy. Recent studies reported an increased risk of bipolar affective disorders in epilepsy, though traditionally considered to be rare. The link between epilepsy and bipolar disorders has also been supported by the effectiveness of several ASMs, such as sodium valproate, carbamazepine, or lamotrigine, for stabilizing mood symptoms (31).

Regarding the neural basis of affective symptoms in epilepsy, abnormalities of serotonin receptors, the involvement of neuroinflammation factors such as interleukin (IL)-1β and IL-6, and the involvement of the hypothalamus-pituitary-adrenocortical system have been proposed (32). More recently, the involvement of opioid receptor desensitization due to repetitive seizures has also been suggested (33). Since as mentioned above mood symptoms in epilepsy can precede the onset of seizures, an elucidation of the neural basis of these symptoms may not only help improve patients’ quality of life; it may also clarify the mechanisms that underlie epileptogenesis. Further progress in this area is expected.

## 6 Epilepsy and cognitive dysfunction

The findings regarding cognitive dysfunction associated with epilepsy are diverse, including those observed in epileptic encephalopathy in children (e.g., Landau-Kleffner syndrome), higher brain dysfunction in common forms of epilepsy such as temporal lobe epilepsy, specific memory deficits such as transient epileptic amnesia, and epilepsy associated with dementia and neurodegenerative diseases. In investigations of individuals with medial temporal lobe epilepsy in which the epileptogenic focus was within the hippocampus or adjacent structures, the so-called material-specific theory was proposed, which states that patients with epilepsy focusing on the language-dominant hemisphere tend to have impaired verbal memory and difficulty in recall, while patients with epilepsy focusing on the nondominant hemisphere show no clear pattern (34). It was also proposed that individuals with medial temporal lobe epilepsy on the language-dominant side are more likely to develop memory deficits after surgical resection for seizure treatment, especially when associated with resection of the posterior hippocampus (35).

Language dysfunction has also been reported to occur with some antiseizure medications such as topiramate and zonisamide, and functional MRI has been used to visualize the drug effects (36). In terms of long-term cognitive function, approx. 60% of older patients with temporal lobe epilepsy and long-term disease durations meet the criteria of mild cognitive impairment (MCI) (37), and compared to older patients with amnesic MCI, they are reported to have milder memory loss and greater language impairment (37). The pattern of medial temporal lobe atrophy was observed to be similar in older patients with temporal lobe epilepsy and those with amnestic MCI (38). Cognitive dysfunction in other epilepsy syndromes, e.g., reduced frontal lobe function in juvenile myoclonic epilepsy (39), has been less frequently reported, and further clarification is warranted. These cognitive or higher brain dysfunctions may also be associated with certain personalities that have been linked to epilepsy syndromes.

Transient epileptic amnesia commonly occurs in men >65 years old, in which patients present anterograde and/or retrograde amnesia mostly upon awakening, and the amnestic symptoms usually return to normal within an hour (40, 41). Antiseizure medications usually well treat and resolve the amnesic episodes in most patients with transient epileptic amnesia. Accelerated long-term forgetting, in which memories cannot be retained after more than a few days, and autobiographical amnesia, in which memories of important life events disappear, have also been proposed to be associated with epileptic amnesia. The accelerated forgetting phenomenon is anterograde amnesia, in which memory consolidation is inhibited, and the memory is retained for up to an hour and does not show abnormalities in usual memory tests, but is forgotten after a few weeks (42). Autobiographical amnesia is a phenomenon in which a person loses memories of important life events that should be unforgettable, e.g., weddings (43). These memory deficits are believed to be epileptic or epilepsy-specific, and the detailed neurobiological mechanisms remain unresolved.

There is already a large body of literature on the bidirectional relationship of epilepsy and dementia (44, 45). The risk of developing epilepsy is more than four times greater in the presence of dementia, and the prevalence of dementia in patients with epilepsy is also higher (46, 47). Data showing that epilepsy hastens cognitive decline (48) and a report of phosphorylated tau in surgical neuropathology of refractory temporal lobe epilepsy in patients >50 years old (49) suggest a close relationship between epilepsy and dementia.

## 7 Epilepsy and neurodevelopmental disorders

The rate of autism spectrum disorder comorbidity in epilepsy is particularly high in tertiary centers at 32% (50) and in population-based statistics at 8.1% (51). Attention-deficit/hyperactivity disorder has shown a greater range at 28%–70% with an odds ratio of 2.7 (52). On the other hand, epilepsy coexists in 13%–38% of patients with autistic spectrum disorders, and 22% have EEG abnormalities without seizures (53). There are some conditions, notably tuberous sclerosis or fragile X syndrome, that are more likely to be associated with epilepsy and autistic spectrum disorders, which are also more likely to be accompanied by intellectual disability. Various factors such as genes, brain structure, metabolism, immunity, and neurotransmitters are thought to be intricately related to the relationship between epilepsy and neurodevelopmental disorders, and the involvement of abnormalities in interneuron function and the balance between excitatory and inhibitory systems has been proposed (54, 55).

## 8 Conclusion

We have briefly reviewed the neuropsychiatric aspects of epilepsy. The idea that the brain plays a certain role in mental disorders has been accepted since the era of Hippocrates (2),. The neuropsychiatric view of mental disorders as disorders of the brain may overlap in some respects with recent biological psychiatry. Unlike the exploration of the brain in most psychiatric disorders for which objective abnormalities have not been established, the consideration of mental and behavioral symptoms in pathological conditions for which objective abnormalities have already been established in the brain from the standpoint of neuropsychiatry, which encompasses neurology, may provide a means to gain new knowledge about the brain. A further development of this field is necessary in order to design and implement better clinical practices involving the human brain.
